# Autoimmune hepatitis as a presenting manifestation of mixed connective tissue disease in a child Case report and review of the literature

**DOI:** 10.1186/s12969-015-0046-4

**Published:** 2015-11-10

**Authors:** Katarina Sedej, Nataša Toplak, Marina Praprotnik, Boštjan Luzar, Jernej Brecelj, Tadej Avčin

**Affiliations:** Department of Allergology, Rheumatology and Clinical Immunology, University Children’s Hospital, University Medical Center, Bohoričeva 20, 1000 Ljubljana, Slovenia; Faculty of Medicine, University of Ljubljana, Ljubljana, Slovenia; Department of Pulmology, University Children’s Hospital, University Medical Center, Ljubljana, Slovenia; Institute of Pathology, Medical Faculty, University of Ljubljana, Ljubljana, Slovenia; Department of Gastroenterology, Hepatology and Nutrition, University Children’s Hospital, Ljubljana, Slovenia

**Keywords:** Mixed connective tissue disease, Autoimmune hepatitis, Child

## Abstract

**Background:**

Liver disease is rare in the course of mixed connective tissue disease. Most commonly liver steatosis or elevated liver function tests are reported and only a few cases of mixed connective tissue disease associated with autoimmune hepatitis were described.

**Case presentation:**

We report a case of an 11-year old boy with hepatitis on admission to the hospital and symptoms and signs of mixed connective tissue disease. Autoimmune hepatitis has been confirmed by liver biopsy.

**Conclusion:**

To the best of our knowledge this is the youngest patient with autoimmune hepatitis as a presenting manifestation of mixed connective tissue disease.

## Background

Mixed connective tissue disease (MCTD) is a systemic rheumatic disease with overlapping selected clinical features of systemic lupus erythematosus (SLE), systemic sclerosis (SS), polymiositis/dermatomyositis and rheumatoid arthritis (RA). High titers of distinct autoantibodies called anti-U1 ribonucleoprotein antibodies (anti-U1 RNP) are characteristic for MCTD. The most common presenting manifestations are polyarthritis, Raynaud’s phenomenon, swollen fingers or hands, myositis, esophageal dysfunction and nonspecific symptoms such as fever, fatigue, arthralgia or myalgia. During the course of the disease pulmonary, renal, cardiac, gastrointestinal and central nervous system manifestations can evolve [1]. Pulmonary involvement is a common complication but is not usually clinically evident early in the course of the disease. In recent years, a growing interest has been focused on severe organ involvement such as interstitial lung disease (ILD) and pulmonary arterial hypertension (PAH) which can evolve during the long-term follow-up and can significantly influence disease prognosis [2].

MCTD is a rare disease in children. Only about 23 % of all MCTD cases appear in childhood. Four different sets of classification criteria for MCTD were developed and none have been evaluated for children. Kasukawa criteria are widely used in pediatric population and require a positive Raynaud’s phenomenon or swollen hands or fingers, a positive test for anti-U1 RNP and at least one sign from two out of three disease categories including SLE, SS or polymyositis. Median age of onset is 11 years. It is more prevalent in female patients with female versus male ratio 6:1 [3].

Autoimmune hepatitis is an immune mediated inflammation of the liver of unknown cause. The disease leads to progressive inflammatory and fibrotic process in the liver [4, 5]. The condition is chronic and characterized by interface hepatitis, hypergammaglobulinaemia and liver-specific serum autoantibodies. The usual age of presentation for type 1 autoimmune hepatitis is from 10 to 20 years or from 45 to 70 years. It is more common in women. Approximately 40 % of patients have concurrent extrahepatic immunological disease [6].

## Case presentation

An 11 year old boy was admitted to our department in January 2012 because of polyarthritis, hepatopathy and Raynaud syndrome. The symptoms first appeared one year before admission when parents noted white fingers on both hands. In September 2011, eight months after the first symptom, the main complaint was fatigue and loss of appetite. He was not gaining weight. No significant fevers above 38 °C were noted, but he was occasionally having mild temperature from 37–38 °C. Gradually his fingers became blue when he was in a cold environment. The change of color became fixed in time and other manifestations developed including headache and joint pain in fingers, wrists, arms, neck, knees and ankles. The worst pain was in fingers in the morning and gradually improved during the day.

Family and his past medical history were unremarkable. At the time of diagnosis he was not receiving medications nor herbal or other products.

On admission he appeared tired and pale. Delayed capillary refill was found, his fingers were livid and cold. Dermatoscopy showed distorted and widened nail fold capillaries. He had swellings of proximal interphalangeal and metacarpophalangeal joints of both hands and swollen knees and ankles. Limited range of motion was found in fingers and thoracolumbar spine. He was not able to touch the ground with fingers. When he bent the distance from his fingertips to the floor was 15 cm. On palpation pain was also induced by performing pressure on patella on both legs and in elbows.

Laboratory investigations showed elevated erythrocyte sedimentation rate, elevated liver function tests, elevated muscle enzymes, elevated lactate dehydrogenase and elevated serum immunoglobulin G, blood cell counts were normal (Table 1). Pancreatic enzymes were also normal. Investigations for metabolic liver diseases and celiac disease were normal. Serological markers for viral etiology of hepatitis were negative (hepatitis A, B and C, Epstein-Barr virus and cytomegalovirus). He was previously vaccinated against Hepatitis B and had positive antibodies against hepatitis B surface antigen.

**Table 1:** Laboratory results before and after treatment. Normal values are presented in legend

	Before treatment	1 month after treatment
ESR (mm/hr)	46	4
E (10^12^/L)	4.41	4.65
Hb (g/L)	115	131
L (10^9^/L)	4.3	11.8
Pl (10^9^/L)	346	306
AST (μkat/L)	2.57	0.78
ALT (μkat/L)	2.38	1.26
gamma GT (μkat/L)	0.41	0.44
CK (μkat/L)	10.10	4.7
LDH(μkat/L)	5.61	4.85
Aldolase (μkat/L)	283	233
IgG (g/L)	27.1	11.9

Legend: *ESR*- erythrocyte sedimentation rate (*N* < 20), *E*- erythrocyte (*N* 4.03–5.29), *Hb*- Hemoglobin (*N* 110–145), *L*- Leukocyte (*N* 3.8–9.8), *Pl*- Platelets (*N* 175–332), *AST*- aspartate aminotransferase (*N* < 0.69), *ALT*- alanine aminotransferase (*N* < 0.52), gamma GT- gamma glutamyltranspeptidase (*N* < 0.37), *CK*- creatine kinase (*N* < 1.86), *LDH*- lactate dehydrogenase (*N* 2.00–5.43), Aldolase (*N* <126), *IgG*- Immunoglobuline G (*N* 6.36–17.01 g/L)

He was evaluated for kidney disease. Urine was normal, protein/creatinine ratio was 7 (normal upper limit is 20). Serum urea and creatinine level and kidney ultrasound were normal.

Immunoserologic tests were positive for antinuclear antigen antibodies (ANA) titer 1:640 and anti-U1 RNP antibodies with a titer 1:256. Liver specific antibodies, anti-ds DNA, antiphospholipid antibodies (anticardiolipin, antiβ 2 glycoprotein) were negative, lupus anticoagulant was not present. Anti-neutrophil cytoplasmic antibodies (ANCA) were also negative. Complement activation was normal.

Muscle function testing including Kendall test and Childhood myositis assessment scale testing, were normal. On skeletal X-ray osteopenia was seen in both hands. Ultrasound of hips showed effusion in his left hip.

Pulmonary function test showed decreased carbon monoxide diffusion capacity of the lungs (DLCO) to 56 % of normal value. Lung CT was normal and there were no signs of ILD.

Abdominal ultrasound showed mild hepatomegaly. Liver biopsy and endoscopic examinations were performed. Gastroscopy revealed *Helicobacter pylori* infection and he received eradication therapy. Colonoscopy did not show any inflammatory changes.

**Fig. 1:**
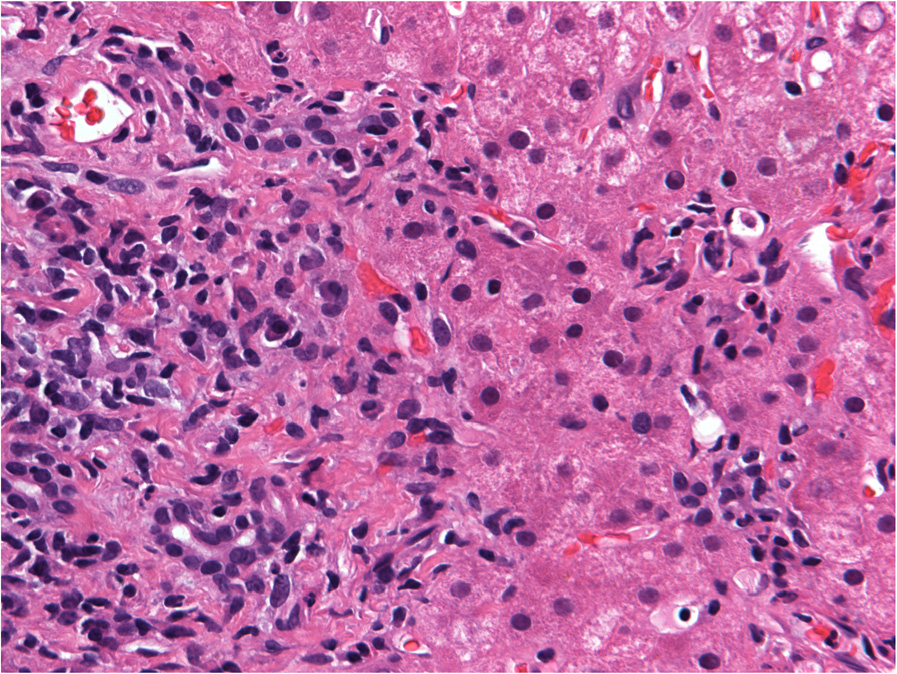
Inflammatory cell infiltrate consisting of lymphocytes and plasma cells is seen extending from the portal tract into the liver parenchyma – mild interface hepatitis. Hematoxylin and eosin, magnification 400x)

**Fig. 2:**
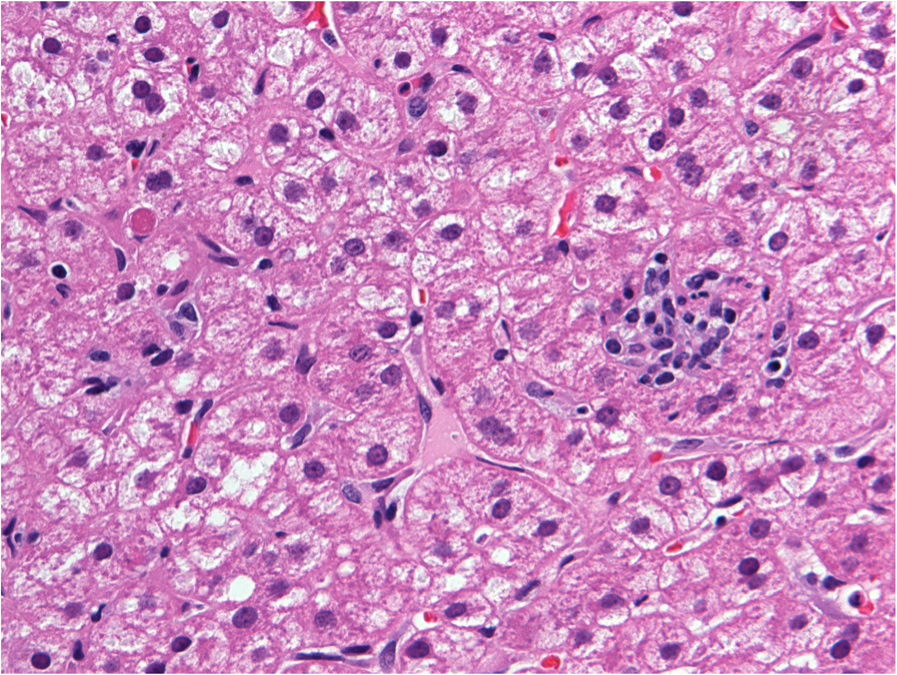
Mild lobular activity and a Councilman body are clearly apparent. Hematoxylin and eosin, magnification 400x)

Liver biopsy revealed a preserved normal architecture. The majority of the portal tracts were slightly expanded and contained mild inflammatory cell infiltrate composed of lymphocytes and plasma cells, extending focally into the liver parenchyma (Fig. 1). Lobular activity was mild and was composed mainly of lymphocytes at the sites of hepatocyte drop-out (Fig. 2). An increased number of apoptotic hepatocytes were also noted. The histological features were consistent with mild autoimmune hepatitis.

Based on the clinical signs and laboratory results our patient fulfilled the Kasukawa diagnostic criteria for MCTD including Raynaud’s syndrome, polyarthritis, reduced DLCO, elevated serum levels of muscle enzymes and positive anti-U1 RNP antibodies. Elevated liver enzymes, positive ANA, high titers of serum immunoglobulin G and negative serology for viral hepatitis indicated a diagnosis of autoimmune hepatitis, which was confirmed by liver biopsy. On revised scoring system for autoimmune hepatitis he scored 16 [7].

The patient was treated with three daily pulses of intravenous methylprednisolone 15 mg/kg BW followed by oral glucocorticosteroid which was slowly tapered. Mycophenolate mofetil and chloroquine were also started. Significant clinical and laboratory improvements were achieved in one month (Table 1). The score on revised scoring system for autoimmune hepatitis fell to 11. Two months after the treatment was started his lung function tests were better but DLCO decreased from 56 % to 49 %. No symptoms or signs of pulmonary disease were present. Heart ultrasound was normal. Six months after treatment was started his DLCO was 69 % and it remained stable during follow up.

Three years after starting therapy he is in remission on methylprednisolone 2 mg once per day, mycophenolate mofetil 750 mg BID and hydroxychloroquine 200 mg per day. He is also taking vitamin D3, calcium and pantoprazole. At the last visit his laboratory results were normal including low level of brain natriuretic peptide (BNP), heart US was normal and lung function tests stable.

The patient and his parents agree with the publication of the case and publication of liver biopsy pictures. Parents signed informed consent.

## Discussion

We presented a case of an 11 year old boy with MCTD and autoimmune hepatitis confirmed by liver biopsy at presentation of the disease. This association is exceedingly rare and to the best of our knowledge only one case of a child with MTCD and autoimmune hepatitis has been previously described [8]. Our patient was successfully treated with methylprednisolone, mycophenolate mofetil and chloroquine. His disease remained inactive on therapy with mycophenolate mofetil and chloroquine when steroids were tapered to low dose. Revised scoring system for autoimmune hepatitis fell from 16 to 11 after treatment was introduced.

Different autoimmune diseases can develop in a single patient. Analysis of 278 patients with autoimmune hepatitis revealed that 40 % of patients were diagnosed with additional autoimmune diseases [6]. The spectrum of hepatic manifestations accompanying rheumatic diseases is also wide and includes chronic active hepatitis, primary biliary cirrhosis, primary sclerosing cholangitis and nodular regenerative hyperplasia [9].

In patients with systemic rheumatic diseases liver can be affected by coexisting autoimmune liver disease like autoimmune hepatitis or primary biliary cirrhosis by direct involvement of the liver parenchyma. Liver damage can also be a consequence of drug toxicity or reactivation of viral hepatitis because of immunosupressive treatment [10].

In the course of autoimmune rheumatic diseases elevation of liver functional tests should prompt further investigations to reveal the reason for liver damage. Hepatic steatosis, nodular regenerative hyperplasia, portal vein obliteration, portal hypertension, features of primary biliary cirrhosis, vascular disorders, granulomatous reactions and rarely portal fibrosis can be found [11]. Liver biopsy is needed to explain the etiology of liver damage.

Only a few cases of female adult patients with mixed connective tissue disease who developed autoimmune hepatitis were previously described [12–15]. A 40-year old woman who was diagnosed with an overlap syndrome of mixed connective tissue disease and Sjoegren’s syndrome was admitted to the hospital with severe cholestatic autoimmune hepatitis and acute liver failure. She responded well to steroid therapy [12]. Another report described a case of a 27-year old woman with MCTD associated with autoimmune hepatitis and thyroiditis [13]. We found only one report of MCTD with autoimmune hepatitis in children. A 16 year old girl with MCTD developed autoimmune hepatitis one year after the disease onset [8].

Long term follow up of a patient with MCTD should include pulmonary function tests on a regular basis and imaging if needed. The survival rate of patients with MCTD depends on lung involvement [2]. An active ILD was detected in 66 % of MCTD patients [16]. Another study in patients with MCTD revealed that 52 % of the patients had abnormal HRCT findings, most commonly lung fibrosis (35 %) [17]. Mortality in patients with normal HRCT was 3.3 %, as compared with 20.8 % in patients with severe lung fibrosis. PAH is another complication of MCTD and has a high mortality rate. Early diagnosis and treatment of PAH are essential. PAH frequency of 3.4 % was found in a cohort of adult patients with MCTD [18]. Doppler-echocardiography is used for diagnostic screening of patients at risk as well as serum pro-BNP and DLCO. The diagnosis needs to be confirmed by right heart catheterization, which is recommended in all patients with suspected PAH [19].

## Conclusions

In conclusion, MCTD and autoimmune hepatitis are both rare diseases in children. To the best of our knowledge, we presented the first report of a child with MCTD and autoimmune hepatitis which was confirmed at the initial presentation. Liver toxicity due to medications and infections should be excluded in such cases and liver biopsy is necessary to establish correct diagnosis.
